# Adiponectin Gene Polymorphism Is Selectively Associated with the Concomitant Presence of Metabolic Syndrome and Essential Hypertension

**DOI:** 10.1371/journal.pone.0019999

**Published:** 2011-05-27

**Authors:** Hsin-Bang Leu, Chia-Min Chung, Shing-Jong Lin, Yuh-Shiun Jong, Wen-Harn Pan, Jaw-Wen Chen

**Affiliations:** 1 Institute of Clinical Medicine, National Yang-Ming University, Taipei, Taiwan, R.O.C.; 2 Cardiovascular Research Center, National Yang-Ming University, Taipei, Taiwan, R.O.C.; 3 Division of Cardiology, Department of Medicine, Taipei Veterans General Hospital, Taipei, Taiwan, R.O.C.; 4 Institutes of Public Health, National Yang-Ming University, Taipei, Taiwan, R.O.C.; 5 Institute of Biochemical Sciences, Academia Sinica, Taipei, Taiwan, R.O.C.; 6 Department of Medical Research and Education, Taipei Veterans General Hospital, Taipei, Taiwan, R.O.C.; 7 Department of Health, Tao-Yuan General Hospital, Tao-Yuan, Taiwan, R.O.C.; 8 Institute of Pharmacology, National Yang-Ming University, Taipei, Taiwan, R.O.C.; Innsbruck Medical University, Austria

## Abstract

**Objective:**

Cardiovascular risk increases with the presence of both metabolic syndrome (MetS) and hypertension (HTN). Although the adiponectin *(ADIPOQ)* gene has been reported to be involved in MetS, its association with HTN remained undetermined. This study aimed to investigate the association of *ADIPOQ* gene with the phenotypes of HTN and MetS.

**Methods:**

A total of 962 participants from 302 families from the Taiwan young-onset hypertension genetic study were enrolled. Plasma adiponectin were measured, and association analysis was conducted by using GEE regression-based method. Another study, of 1448 unrelated participants, was conducted to replicate the association between *ADIPOQ* gene and variable phenotypes of MetS with or without HTN.

**Results:**

Among 962 subjects from family samples, the lowest plasma adiponectin value was observed in MetS with HTN component (9.3±0.47 µg/ml) compared with hypertensives (13.4±0.74 µg /ml) or MetS without HTN (11.9±0.60 µg/ml, P<0.05). The SNP rs1501299 (G276T) in *ADIPOQ* gene was found associated with the presence of HTN in MetS (odds ratio for GG+GT vs. TT = 2.46; 95% CI: 1.14-5.3, p = 0.02), but not rs2241766 (T45G). No association of *ADIPOQ* gene with HTN alone or MetS without HTN was observed. The significant association of the SNP rs1501299 (G276T) with the phenotype of presence of HTN in MetS was confirmed (odds ratio for GG+GT vs. TT = 2.15; 95% CI: 1.1–4.3) in the replication study.

**Conclusions:**

*ADIPOQ* genetic variants were selectively and specifically associated with the concomitant presence of MetS and HTN, suggesting potential genetic linkage between MetS and HTN.

## Introduction

It is suggested that metabolic syndrome (MetS), the presence of various metabolic and cardiovascular risk factors including insulin resistance, glucose intolerance, central obesity, elevated blood pressure, and dyslipidemia, may lead to poor clinical outcomes with increased cardiovascular morbidity and early death [1], [2]. However, not all components of MetS contribute equally to the risk. Hypertension (HTN), rather than other risk factors, has been reported to be independently associated with an increased cardiovascular risk in subjects with MetS [3]. Hypertensive patients with MetS have double the cardiovascular risk compared to those without [4]. Thus, the presence of hypertension components of MetS might imply a potential clinical sub-phenotype with higher risk. However, it was not known whether there is pathophysiological and genetic background contributing to the HTN component of MetS. Previous studies which investigated the heritability of MetS demonstrated there were two independent clusters of composing factors in MetS – lipids, glucose, and obesity in one group and blood pressure alone in another group, suggesting possible distinct genetic background for MetS containing HTN component [5]. Recently, with genome-wide scanning, several novel genetic loci have been found related to the clinical presentation of MetS in different populations [6], [7], but the genetic linkage between essential hypertension and MetS remained undetermined.

Adiponectin is an adipose tissue-derived cytokine with anti-inflammatory and anti-atherogenic properties which was linked to central obesity and proposed as major contributor to MetS in addition to insulin resistance [8]. Reduced plasma adiponectin levels were observed in patients with obesity, diabetes, and coronary artery disease [9]. Though the presence of MetS has been linked to decreased plasma adiponectin values, the role of adiponectin (*ADIPOQ*) gene in determining MetS components especially HTN [9], remained undetermined [10]. Furthermore, reduced plasma adiponectin level was observed among young healthy participants with hypertensive parents [11], suggesting genetic association with *ADIPOQ* may contribute to the phenotype of HTN. In addition to clinical observation, knockout mice lacking *ADIPOQ* gene also exhibited HTN, hypertriglyceridemia, and hyperglycemia, the features of MetS [12]. Taken all together, this evidence implies it is possible that the *ADIPOQ* gene might be linked to the presence of MetS and HTN. The present study was conducted to investigate whether *ADIPOQ* genetic variants are associated with the phenotype of the presence of HTN among MetS. This study was conducted by a family-based approach, and subsequently verified by another independent sample.

## Methods

### Subjects and phenotype definition

#### Family-based association study

**Figure 1:**
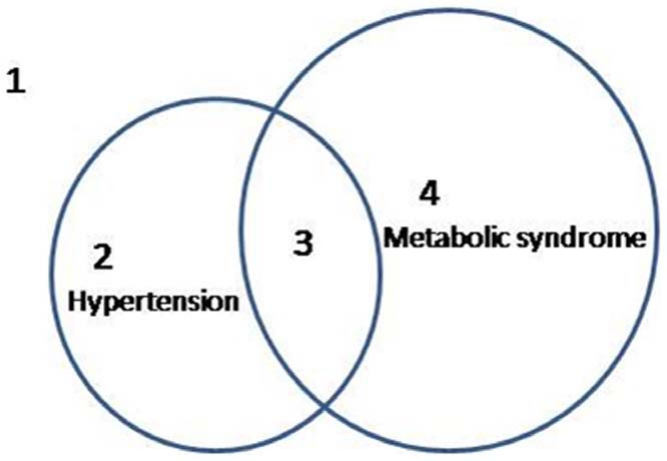
The compositions of the study subjects in both family association study and case-control study, which included 1, sub-phenotype 1, the subjects with neither hypertension nor metabolic syndrome; 2, sub-phenotype 2, the subjects with hypertension but no metabolic syndrome; 3, sub-phenotype 3, the subjects with both hypertension and metabolic syndrome; 4, sub-phenotype 4, the subjects with metabolic syndrome but no hypertension.

Study subjects from the family association study were enrolled from the Taiwan young-onset hypertension genetic study [13] and young hypertension probands were identified from four community hospitals located in northwest Taiwan. For each identified proband, family members including their parents, affected or unaffected sib pairs were invited for a BP screening and enrolled into this study. The family structure comprised two generations and was a nuclear-family format. A total of 962 subjects, 495 men and 467 women from 302 nuclear families, were enrolled in this study. Both parents of probands were recruited in 23.1% of the families, and one parent in 27.2% of the families. Four distinct phenotypes, of (1) normotensive and non-metabolic syndrome; (2) HTN without MetS; (3) MetS with HTN, and (4) MetS without HTN, were clearly identified in this family association study database (**Figure 1**). The diagnosis of HTN was defined as systolic blood pressure >140 mmHg and/or diastolic blood pressure >90 mmHg or currently taking at least one antihypertensive medication. Exclusion criteria were the presence of any of secondary causes of hypertension such as chronic renal disease, renal arterial stenosis, primary aldosteronism, coarctation of the aorta, thyroid disorders, Cushing syndrome, and pheochromocytoma through extensive clinical examinations and investigations (including blood chemistry, renal function tests, endocrine examination, and abdominal sonogram). Participants who fulfilled at least three of the following five criteria of MetS were defined as “affected” status for MetS according to the ATPIII criteria. The five criteria included (1) blood pressure ≥ 130/85 mmHg, (2) fasting plasma glucose level >110 mg/dL, (3) hypertriglyceridemia with triglyceride level ≥150 mg/dL, (4) high-density lipoprotein-cholesterol (HDL-C) level <1.0 mmol/l in men or <1.3 mmol/l in women, and (5) central obesity with waist circumference >90 cm in men or >80 cm in women. The definition of central obesity has been modified for the Asian population [14]. Participants were divided into variable phenotypes of HTN and MetS.

#### Replication study

In order to replicate the finding found in the family-based study, we conducted another study including 1448 unrelated subjects without cardiovascular events to confirm the above study' results. The replication study included an unrelated 706 males and 742 females, who were grouped into subtypes of HTN and MetS according to the above family based association study design. The study cohort was selected from the CardioVascular Disease risk FACtors Two-township Study (CVDFACTS) [15], [16]. Briefly, the CVDFACTS was a community-based follow-up study investigating the cardiovascular disease occurrence and risk factors in Taiwan since 1989. Five villages with more than 1000 people and population density greater than 200 people per square kilometer were randomly selected from Chu-Dong (northwest Taiwan) and Pu-Tzu (southwest Taiwan) areas. Information about participants' lifestyle, risk factors, history of cardiovascular disease, urine and blood chemistry data was collected. The study protocol was approved by the Human Investigation Committee of the Institute of Biochemical Science, Academia Sinca, Taiwan, and all subjects gave written informed consent.

### DNA preparation and genotyping

Blood pressure, pulse, height, and weight were measured according to the standardized protocol established for the Nutrition and Health Survey in Taiwan [17]. Forearm venous blood samples were drawn from each patient or participant by venipuncture after an overnight fast of 10–12 hours. The buffy coat (the white cell layer) was pipetted into a vial and frozen at −70°C. Genomic DNA of the probands and their relatives were isolated from collected peripheral lymphocytes by the phenol/chloroform extraction method. The reason why we selected these three SNPs is that based on previous findings. The two SNP markers rs2241766 (T45G) and rs15011299 (G276T), are the common informative SNP markers in Asian populations. Although many SNP markers in *ADIPOQ* have been studied, rs2241766 (T45G) and rs15011299 (G276T) were reported to have a strong association with MetS in Japanese population [18]. Furthermore, they are especially considered for the MetS-associated *ADIPOQ* SNPs in Asian people among five different populations [19]. In addition to SNPs rs2241766 (T45G) and rs15011299 (G276T), SNP (I164T) [20] has been found to be associated with MetS and coronary artery disease in Japanese population. Therefore, after considering the Asian origin of our study population, these three SNPs were selected for genotyping to test the association between *ADIPOQ* gene and phenotypes in the current study. The primary genotyping technique was TaqMan SNP allelic discrimination using an ABI 7900HT (Applied Biosystems, Foster City, CA) as previously described.

### Measurement of metabolic profiles and plasma adiponectin level

Metabolic profiles, including total cholesterol, HDL-C, triglyceride, and other biochemical parameters were measured using a Hitachi 7600-310 autoanalyzer (Hitachi Ltd., Tokyo, Japan). Plasma concentration of high-molecular-weight adiponectin were determined by a sandwich enzyme-linked immunosorbent assay system (Otsuka Pharmaceutical Co., Tokushima, Japan) as described previously [21].

### Statistical analysis

All data were expressed as mean ± SEM if normally distributed or as median (range) otherwise. Parametric continuous data between groups were compared by an unpaired Student's *t*-test and ANOVA test. Categorical data between groups were compared with the chi-square test or Fisher's exact test. The comparisons of genetic association with *ADIPOQ* variants were used to evaluate the association of the presence of each phenotype compared with the remaining subjects and *ADIPOQ* SNP markers. Because family members are not independent with respect to each other, family structure was adjusted using a generalized estimating equation (GEE) (GEE model, Proc GENMOD using SAS statistical software) [22], taking into account familial dependence of the observations to estimate the association of *ADIPOQ* genetic variant between these distinct phenotypes of HTN and MetS. Parameters including age, sex, body mass index, glucose, waist circumference, total cholesterol, triglycerides and HDL-C were added for adjustment. In addition, the association of MetS parameters and *ADIPOQ* SNP were assessed by linear regression to determine the genetic association of *ADIPOQ* variants and quantitative traits of MetS.

To verify the association between ADIPOQ SNP and phenotypes, we utilized data from the independent cohort study to perform regression analysis on SNPs in the *ADIPOQ* gene with respect to variable phenotypes of interest. Age, gender, blood pressure, and metabolic profiles were included as covariates. Parametric continuous data between groups were compared by unpaired Student's *t*-test and ANOVA test. Categorical data between groups were compared with the chi-square test or Fisher's exact test. In the family association study, G allele of rs15101299 (G276T) of the *ADIPOQ* gene was significantly associated with the phenotype of metabolic syndrome with HTN component under the dominant model. Therefore, the replication study tested the genetic association of distinct phenotype compared with other remaining subjects using TT genotype as a referent. All these statistical analysis were performed using SAS for Window software (version 9.1).

## Results

### Basic characteristics of the family study participants

A comparison of characteristics of variable phenotypes of MetS with or without HTN in the young-onset HTN families is shown in **Table 1**. Compared with the normotensive participants, hypertensive participants had significantly higher blood pressure, larger BMI, and increased waist circumference. Furthermore, hypertensive subjects had higher serum triglyceride and cholesterol levels, and lower HDL-C and adiponectin levels, than the normotensive participants. Among 962 subjects from family samples, the lowest plasma adiponectin value was observed in MetS with HTN component (9.3±0.47 µg/ml) compared with that in hypertensives (13.4±0.74 µg/ml) or MetS without HTN (11.9±0.60 µg/ml, P<0.05). The association was still significant after adjustment for metabolic profiles and BMI, and was also consistent in both men and women when analyzed separately.

**Table 1:** Baseline characteristics of all family subjects.

	MetS (−)		MetS (+)	
Trait	HTN (−)(n = 446)	HTN (+)(n = 159)	HTN (−)(n = 165)	HTN(+)(n = 192)
Age, years	46.7±0.8† ‡ §	41.4±0.7* †	57.5±1.0* ‡ §	41.7±0.6* †
Male, n (%)	215 (49)	90 (57)	65(39)	125 (65)
BMI, kg/m^2^	23.5±0.1† ‡ §	25.4±0.3* † §	27.1±0.3* ‡ §	28.3±0.3* † ‡
Waist circumference, cm	79±0.5† ‡ §	83.1±0.7* † §	90.4±0.7* ‡	92.9±0.6* ‡
Blood pressure, mmHg				
Systolic	119.4±0.8† ‡ §	140.3±1.5*	137.7±1.4*	140.1±1.4*
Diastolic	74.3±0.6† ‡ §	89. ±1.0* † §	83.3±0.9* ‡ §	90.5±1.0* † ‡
Metabolic profiles				
Cholesterol, mg/dl	192.9±2.0† §	195.7±3.1† §	209.5±3.5* ‡	215.5±3.9* ‡
HDL-C, mg/dl	53.9±.7† §	52.2±0.9† §	42.0±0.8* ‡	41.1±0.7* ‡
LDL-C, mg/dl	119.8±1.8	125.6±2.9	122.7±3.1	127.7±3.4
Triglycerides, mg/dl	110.9±3.4† §	116.5±5.5† ‡	235.4±11.1* ‡	241.3±12.2* ‡
Glucose, mg/dl	96.7±1.3† §	98.2±1.4† §	142.9±6.9* ‡ §	127.4±4.5* † ‡
Adiponectin, µg/ml	15.9±0.5† ‡ §	11.9±0.6* §	13.4±0.7* §	9.3±0.5* † ‡
*ADIPOQ* genotype				
rs1501299 (G276T)				
TT, n (%)	40 (8.9)	14 (8.8)	16 (9.8)	7 (3.8)
GT, n (%)	178 (39.9)	60 (37.7)	56 (33.7)	68 (35.6)
GG, n (%)	228 (51.1)	85 (53.4)	93 (56.4)	117 (60.6)
rs2241766 (T45G)				
GG, n (%)	32 (7.2)	15 (9.4)	12 (7.2)	19 (9.9)
TG, n (%)	181 (40.6)	70 (44)	75 (45.5)	81 (42.1)
TT, n (%)	233 (52.2)	74 (46.5)	78 (47.3)	92 (47.9)

Data are mean ± SEM or number (%); HTN indicates hypertension; MetS, metabolic syndrome; BMI, indicates body mass index; HDL-C, high-density lipoprotein cholesterol; LDL-C, low-density lipoprotein cholesterol.

*P< 0.001 vs.MetS(-)HTN(−);

†P< 0.001 vs. MetS(+)HTN(−);

‡P< 0.001 vs. MetS(−)HTN(+);

§P< 0.001 vs. MetS(+)HTN(+).

### SNP polymorphisms of adiponectin gene in study population

Among the three SNP markers of the adiponectin gene used in the young-onset HTN family study, the I164T polymorphism, which was previously reported in the Japanese population, was not found to be informative in our study participants. In our study subjects, the minor allele frequencies of rs2241766 (T45G) T>G and rs1501299 (G276T) G>T were 29.2% and 27.2%, respectively. Allele frequencies of both markers did not show any significant deviation from those expected according to Hardy-Weinberg equilibrium in either population. Only the SNP rs1501299 (G276T) was found associated with the presence of HTN in MetS after adjusting for age gender, waist circumference and metabolic profile using a GEE analysis under a dominant model (odds ratio for GG+GT vs. TT = 2.46; 95% CI: 1.14-5.3, p = 0.02) (**Table 2**). No association of *ADIPOQ* with merely HTN or MetS without hypertension was observed. **Table 3** shows the regression analysis of individual components and plasma adiponectin value, and *ADIPOQ* gene variants. There was no significant association between *ADIPOQ* genotypes and plasma adiponectin values as well as MetS parameters in our study.

**Table 2:** GEE analysis for association of adiponecin variant and trait in variable phenotypes of hypertension and metabolic syndrome among family subjects.

Phenotype		Genotype	OR (95%CI)
a **MetS(-)**	**HTN(-)**		
rs1501299 (G276T)		TT	1
		GT	0.87 (0.51–1.47)
		GG	0.66 (0.4–1.11
		TT	1
		GG+GT	0.74 (0.45–1.22)
rs2241766 (T45G)		GG	1
		TG	0.91 (0.53–1.55)
		TT	1.05 (0.62–1.79)
		GG	1
		TT+TG	0.98 (0.59–1.64)
b **MetS (−)**	**HTN (+)**		
rs1501299 (G276T)		TT	1
		GT	0.90 (0.47–1.74)
		GG	0.93 (0.49–1.75)
		TT	1
		GG+GT	0.92 (0.49–1.7)
rs2241766 (T45G)		GG	1
		TG	0.87 (0.46–1.68)
		TT	0.78 (0.41–1.49)
		GG	1
		TT+TG	0.82 (0.44–1.53)
c **MetS (+)**	**HTN (−)**		
rs1501299 (G276T)		TT	1
		GT	0.70 (0.35–1.43)
		GG	0.9 (0.46–1.78)
		TT	1
		GG+GT	0.82 (0.42–1.59)
rs2241766 (T45G)		GG	1
		TG	1.62 (0.73–3.6)
		TT	1.47 (0.67–3.26)
		GG	1
		TT+TG	1.54 (0.71–3.33)
d **MetS(+)**	**HTN (+)**		
rs1501299 (G276T)		TT	1
		GT	2.11 (0.95–4.7)
		GG	**2.74 (1.25**–**5.98)** *
		TT	1
		GG+GT	**2.46 (1.14**–**5.3)** *
rs2241766 (T45G)		GG	1
		TG	0.89 (0.48–1.65)
		TT	0.90 (0.79–1.65)
		GG	1
		TT+TG	1.45 (0.93–2.06)

All estimates were analyzed using generalized estimating equation (GEE) model adjusted for age, sex, body mass index, glucose, blood pressure, waist circumference, triglycerides, and HDL-choleserol.

*p<0.05 adjusting for age and gender. HTN indicates hypertension; MetS, metabolic syndrome.

a If the presence of phenotype of MetS(−)HTN(−) compared with other remaining subjects.

b If the presence of phenotype of MetS(−)HTN(+) compared with other remaining subjects.

c If the presence of phenotype of MetS(+)HTN(−) compared with other remaining subjects.

d If the presence of phenotype of (+)HTN(+) compared with other remaining subjects.

**Table 3:** Associations between MetS trait and ADIPOQ genetic variants.

	rs1501299 (G276T)			Risk allele (Genotypes)		β(P)	
	GG	TG	TT	G(G/T)	Model 1^a^	Model 2*^b^*	Model 3*^c^*
**HDL-C, mg/dl**	48.66±0.622	49.26±0.735	49.59±1.197		−1.599(0.09)	−4.712(0.36)	−1.267(0.294)
**Glucose, mg/dl**	109.06±2.103	114.2±3.343	108.72±4.637		−1.187(0.769)	9.272(0.337)	−4.542(0.377)
**Triglycerides, mg/dl**	162.32±5.801	166.46±8.09	140.55±10.496		−1.394(0.913)	−2.758(0.928)	−1.485(0.927)
**Systolic BP, mmHg**	130.35±0.923	130.5±1.107	128.72±2.22		−0.336(0.849)	2.109(0.617)	−1.141(0.611)
**Diastolic BP, mmHg**	81.74±0.609	82.66±0.8	79.7±1.39		−1.064(0.374)	1.653(0.565)	−2.194(0.150)
**WC, cm**	84.49±0.48	84.46±0.6	84.06±1.38		0.356(0.672)	1.551(0.443)	0.140(0.896)
**Adiponectin, µg/ml**	13.54±0.417	13.27±0.516	12.85±1.047		0.269(0.640)	0.420(0.758)	0.323(0.663)

	rs2241766 (T45G)			Risk allele (Genotypes)		β(P)	
	TT	TG	GG	T(T/G)	Model 1^a^	Model 2*^b^*	Model 3*^c^*
**HDL-C, mg/dl**	48.94±1.56	49.35±0.63	48.42±0.73		−0.159(0.763)	−3.045(0.134)	0.780(0.514)
**Glucose, mg/dl**	109.15±7.08	111.13±2.23	109.30±2.68		0.726(0.859)	2.949(0.748)	0.237(0.965)
**Triglycerides, mg/dl**	152.5±13.93	164.4±7.32	167.57±7.24		5.847(0.415)	19.839(0.239)	0.881(0.963)
**Systolic BP, mmHg**	135.16±2.91	128.6±0.97	131.34±0.99		−3.142(0.064)	−7.532(0.073)	−0.2.835(0.204)
**Diastolic BP, mmHg**	84.45±2.0	79.9±0.62	83.2±0.72		−1.975(0.088)	−3.805(0.142)	−2.105(0.167)
**WC, cm**	86.89±1.27	83.76±0.54	85.06±0.54		−0.245(0.763)	−2.517(0.164)	0.448(0.674)
**Adiponectin, µg/ml**	16.09±1.38	13.16±0.43	13.39±0.50		−0.791(0.158)	−1.347(0.282)	−0.902(0.222)

Data are mean ± SEM or β(P). HDL-C indicates high-density lipoprotein cholesterol; WC, waist circumference; BP, blood pressure.

All estimates were analyzed using liner regression model, and adjustments were made for age, sex, body mass index, glucose, blood pressure, waist circumference, triglycerides, and HDL-choleserol.

a Model 1: additive model; ^b^Model 2: dominant model; ^c^Model 3: recessive model.

### Replication of the association between *ADIPOQ* and the concomitant presence of HTN and MetS in case-control study

In the subsequent verification study with 1448 unrelated subjects (**Table 4**), the association of G allele of *ADIOPQ* SNP rs1501299 (G276T) between the phenotype of MetS with HTN in family cohort was confirmed (odds ratio for GG+GT *vs.* TT: 2.15; 95% CI: 1.1–4.3) (**Table 5**). There was no association of *ADIPOQ* genetic variant with HTN alone or with MetS without HTN. Therefore, rs1501299 (G276T) polymorphism of *ADIPOQ* gene may play a significant role in the genetic connection between HTN and MetS **(** **Table 5** **).**

**Table 4:** Baseline characteristics of case-control designed replication study.

	MetS(−)		MetS(+)	
	HTN (−)(n = 667)	HTN (+)(n = 246)	HTN (−)(n = 240)	HTN(+)(n = 290)
Age, years	53.3±0.4 † ‡ §	57.4±0.7*	56.8±0.6*	57.8±0.6*
Male, n (%)	338(50.7)	150(61)	82 (33.5)	136(47)
BMI, kg/m^2^	23.1±0.1 † §	23.5±0.2 † §	26.4±0.2* ‡	26.4±0.2* ‡
Waist circumference, cm	78.1±0.3 † §	79.7±0.5 † §	87.9±0.5* ‡	87.9±0.5* ‡
Blood pressure, mmHg				
Systolic	111.4±0.4 † ‡ §	139.2±0.9* † §	114.8±0.6* ‡ §	141.8±0.9* †
Diastolic	71.5±0.3 † ‡ §	86.4±0.6* †	73.2±0.5* ‡ §	86.7±0.6* †
Metabolic profiles				
Cholesterol, mg/dl	194.0±1.6 † §	195.8±2.6 † §	215.6±2.9* ‡	214.5±2.5* ‡
HDL-C, mg/dl	46.0±0.7 † §	48.6±0.8 † §	35.5±0.5* ‡	37.0±0.6* ‡
LDL-C, mg/dl	124.8±2.1 †	135.3±8.3	144.9±8.4*	143.3±7.5
Triglycerides, mg/dl	86.5±1.6 † §	83.6±2.3 † §	191.7±8.8* ‡	158.9±3.3* ‡
Glucose, mg/dl	92.1±1.5 † §	93.0±2.5 † §	111.7±4.6* ‡	107.5±4.6* ‡

Data are mean ± SEM or number (%); HTN indicates hypertension; MetS, metabolic syndrome; BMI, indicates body mass index; HDL-C, high density lipoprotein cholesterol; LDL-C, low density lipoprotein cholesterol.

*P< 0.001 vs. MetS(−) HTN(−);

†*P*< 0.001 vs. MetS(+) HTN(−);

‡*P*< 0.001 vs. MetS(−) HTN(+);

§*P*< 0.001 vs. MetS(+) HTN(+).

**Table 5:** The association of *ADIPOQ* variants and variable subtypes of hypertension and metabolic syndrome among study subjects in replication study.

Trait		No. of participants	Genotype at rs1501299 (G276T)			OR for GG+GT vs. TT (95% CI)
			GG	GT	TT	
			No. of subjects			
**MetS (−)**	**HTN(−)** a	667	366 (54.8)	261 (39.1)	40 (5.9)	0.72 (0.45–1.15)
**MetS (−)**	**HTN(+)** b	246	130 (52.8)	103 (41.9)	13 (5.3)	0.96 (0.52–1.78)
**MetS(+)**	**HTN(−)** c	240	133 (55.4)	95 (39.6)	12 (5)	1.03 (0.55–1.95)
**MetS(+)**	**HTN(+)** d	**290**	**202 (69.6)**	**79 (27.2)**	**9 (3.1)**	**2.15 (1.1**–**4.3)** *

Trait		No. of participants	Genotype at rs2241766 (T45G)			OR for TT+TG vs. GG (95% CI)
			TT	TG	GG	
			No of subjects			
**MetS (−)**	**HTN(−)** a	667	319 (47.8)	300 (45)	48 (7.2)	1.14 (0.77–1.68)
**MetS (−)**	**HTN(+)** b	246	127 (51.6)	98 (39.8)	21 (8.5)	0.87 (0.53–1.43)
**MetS(+)**	**HTN(−)** c	240	120 (50)	101 (42.1)	19 (7.9)	0.96 (0.58–1.61)
**MetS(+)**	**HTN(+)** d	290	144 (49.7)	123 (42.4)	23 (7.9)	0.96 (0.59–1.55)

All estimates were analyzed by logistic regression model adjusted for age, gender, body mass index, blood pressure, glucose, waist circumference, triglycerides, and HDL-cholesterol.

HTN indicates hypertension; MetS, metabolic syndrome;

*P< 0.05

a If the presence of phenotype of MetS(−) HTN(−) compared with other remaining subjects

b If the presence of phenotype of MetS(−) HTN(+) compared with other remaining subjects

c If the presence of phenotype of MetS(+) HTN(−) compared with other remaining subjects

d If the presence of phenotype of MetS(+) HTN(+) compared with other remaining subjects.

## Discussion

Different from the previous studies regarding the genetics of MetS as a whole, the present study is the first one to demonstrate the association of *ADIPOQ* gene rs1501299 (G276T) mutation with the concomitant presence of MetS and HTN. Such association could not be seen in subjects with HTN alone or in normotensive subjects with MetS, suggesting a selective genetic linkage of *ADIPOQ* gene between MetS and essential HTN. HTN and MetS might overlap partially with each other as a specific clinical sub-phenotype with common genetic background.

Our current findings especially elucidate the genetic background of subjects of MetS with HTN, which is different from and could not be applied to all the patients with MetS or HTN. Although adiponectin is considered linked to MetS, a study with genotyping 53 SNPs of the *ADIPOQ* gene failed to find any correlation between *ADIPOQ* genetic variants and parameters of MetS, suggesting the limited impacts of the *ADIPOQ* gene on individual parameters of MetS [10]. Recently, a genome wide association study (GWAS) showed no association between plasma adiponectin and genetic loci associated with MetS parameters, suggesting genetically determined adiponectin probably does not modulate MetS directly [23]. Actually, previous clinical observation demonstrated that MetS could be dissected into two major components: glucose/obesity/lipid as one cluster, and blood pressure as the other one [5], [24]. Subsequent heritability studies of MetS also showed these two groups, lipid/glucose/obesity and high blood pressure, significantly contributed the genetic background of MetS [5], [25], indicating the heterogeneous nature of MetS and possible different distinct pathogenesis between MetS with and without HTN [5]. Our current study demonstrated *ADIPOQ* associated with MetS with HTN component instead of HTN alone or metabolic MetS without HTN component, suggesting that MetS should be seen as clusters of metabolic abnormalities and *ADIPOQ* may contribute genetically in some clusters of MetS. This could explain why there was inconsistent association observed between *ADIPOQ* and individual MetS parameters in previous studies, and help to clarify previous observations on the connection of *ADIPOQ* with MetS. Future study is indicated to investigate the individual genetic background and clinical significance in different subtypes of MetS rather than in MetS as a whole.

Although more than 25 different genes have been identified to connect to essential HTN either by linkage or association studies, the replication studies have not shown consistent associations [26]. The WTCCC GWAS also failed to define any genetic signal associated with HTN, indicating heterogeneity in the nature of HTN [27]. Therefore, searching for clinically relevant subtypes of HTN such as the concomitant presence of MetS and HTN may be mandatory to dissect this complex disease. In the current study, we demonstrated the association of favoring G allele of rs1501299 (G276T) polymorphism with the presence of HTN in MetS. However, the overall reported associations of rs1501299 (G276T) polymorphism and cardio-metabolic disease were diverse. It has been reported that the T allele of rs1501299 (G276T) polymorphism associated with increased insulin resistance and type 2 diabetes [28], [29]. On the other hand, subjects carrying the G allele of rs1501299 (G276T) polymorphism were reported to have higher BMI, insulin resistance, increased blood pressure, and left ventricle hypertrophy [30], providing direct evidence that the G allele of rs1501299 (G276T) may lead to target organ damage. It is also in accordance with our finding that *ADIPOQ* gene might contribute to the genetic association with the phenotype of presence of HTN in MetS. Future study is indicated to evaluate the direct impacts of the G allele of rs1501299 (G276T) polymorphism on clinical outcomes in HTN patients.

Our current study demonstrated that the SNP variant in *ADIPOQ* gene could be associated with MetS including HTN component rather than with HTN alone or with MetS excluding HTN component, and that the former phenotype had the lowest plasma adiponectin value, suggesting that MetS could be seen as clusters of metabolic abnormalities, and that *ADIPOQ* may selectively contribute to some clusters of MetS, especially those with HTN. Our findings may provide the rationale for the inconsistent association between *ADIPOQ* and individual MetS parameters in previous studies, and help to clarify the previous observations about the connections between *ADIPOQ* and MetS.

There are some limitations in our study. First, there was no significant association between plasma adiponectin value and selected *ADIPOQ* SNP markers. It has been noted that previous studies focused on the association between plasma adiponectin level and *ADIPOQ* gene variants were not consistent and occasionally discrepant [20], [31]. Similar to our findings, no significant associations between plasma adiponectin level and *ADIPOQ* variants rs2241766 (T45G) and rs1501299 (G276T) were reported [20]. There are several potential reasons for the inconsistent association between SNP and plasma adiponectin value. First, rs1501299 (G276T) is in intron 2 of the *ADIPOQ* gene and does not have a known function. It is probably a marker of some other variant affecting adiponectin expression. Furthermore, rs1501299 (G276T) is in a linkage disequilibrium block encompassing most of the *ADIPOQ* gene, but whether and how far such block extends beyond the gene boundaries remain to be determined [32]. Second, even considering the SNP markers with *ADIPOQ* gene, other gene loci such as 14q13 have been reported to affect plasma adiponectin value and play a much bigger role [32]. Recently, GWAS for genetic markers in determining plasma adiponectin value in Asian population reported that genetic variants in *CDH13* on chromosome 16, but not genetic variants in the *ADIPOQ* gene, influence adiponectin levels in Korean adults.[33] However, another GWAS using plasma adiponectin as a quantitative trait demonstrated the *ADIPOQ* gene as the only major gene for plasma adiponectin in Caucasian population.[23] Racial and dietary differences between Caucasian and Asian populations are also proposed for the differences in GWAS findings. Furthermore, it has been indicated that in addition to genetic effect, plasma adiponectin level may be significantly modified by cardiovascular risk factors and life style [28]. Accordingly, the above factors may differentially modify the association of plasma adiponectin level with *ADIPOQ* gene variants in different ethnic cohorts. Third, the subjects in this cohort study were enrolled from community a few years ago. The definite medication information could not be identified in detail at that time. Fourth, because of the enrollment limitation in the family-based association study, the case number in each phenotype was unequal, and only the subgroup of normotensive and non-MetS, the largest subgroup, could achieve statistical power up to 91.72% at alpha = 0.05. The statistical powers in other subgroups were around 0.6–0.7 at alpha = 0.05, probably due to inadequate number of cases. However, although the case number may give less satisfactory power for statistics in the family-based study, the association between the SNP variant in *ADIPOQ* gene and MetS with HTN component could be replicated in another large independent cohort, indicating a significant association. Finally, currently selected SNP markers may be limited and inadequate to demonstrate the genetic connection of plasma adiopnectin value. Further larger-scale high-throughput genotyping has being carried out to investigate the association between genetic variants and plasma adiponectin value.

In conclusion, the present study demonstrated the association of both plasma adiponectin level and the variants of *ADIPOQ* gene with the presence of essential HTN patients with MetS, suggesting a selective genetic linkage of adiponectin between essential HTN and MetS. Our findings suggest that HTN and MetS might overlap partially with each other as a specific clinical sub-phenotype with common genetic background.

## References

[1] Reaven GM (1998). Banting lecture 1988. Role of insulin resistance in human disease.. Diabetes.

[2] Rutter MK, Meigs JB, Sullivan LM, D'Agostino RB, Wilson PW (2005). Insulin resistance, the metabolic syndrome, and incident cardiovascular events in the Framingham Offspring Study.. Diabetes.

[3] Mancia G, Bombelli M, Corrao G, Facchetti R, Madotto F (2007). Metabolic syndrome in the Pressioni Arteriose Monitorate E Loro Associazioni (PAMELA) study: daily life blood pressure, cardiac damage, and prognosis.. Hypertension.

[4] Schillaci G, Pirro M, Vaudo G, Gemelli F, Marchesi S (2004). Prognostic value of the metabolic syndrome in essential hypertension.. J Am Coll Cardiol.

[5] Lin HF, Boden-Albala B, Juo SH, Park N, Rundek T (2005). Heritabilities of the metabolic syndrome and its components in the Northern Manhattan Family Study.. Diabetologia.

[6] An P, Freedman BI, Hanis CL, Chen YD, Weder AB (2005). Genome-wide linkage scans for fasting glucose, insulin, and insulin resistance in the National Heart, Lung, and Blood Institute Family Blood Pressure Program: evidence of linkages to chromosome 7q36 and 19q13 from meta-analysis.. Diabetes.

[7] Ng MC, So WY, Lam VK, Cockram CS, Bell GI (2004). Genome-wide scan for metabolic syndrome and related quantitative traits in Hong Kong Chinese and confirmation of a susceptibility locus on chromosome 1q21-q25.. Diabetes.

[8] Okamoto Y, Kihara S, Funahashi T, Matsuzawa Y, Libby P (2006). Adiponectin: a key adipocytokine in metabolic syndrome.. Clin Sci (Lond).

[9] Yang WS, Chuang LM (2006). Human genetics of adiponectin in the metabolic syndrome.. J Mol Med.

[10] Heid IM, Wagner SA, Gohlke H, Iglseder B, Mueller JC (2006). Genetic architecture of the APM1 gene and its influence on adiponectin plasma levels and parameters of the metabolic syndrome in 1,727 healthy Caucasians.. Diabetes.

[11] Furuhashi M, Ura N, Higashiura K, Miyazaki Y, Murakami H (2005). Low adiponectin level in young normotensive men with a family history of essential hypertension.. Hypertens Res.

[12] Kubota N, Terauchi Y, Yamauchi T, Kubota T, Moroi M (2002). Disruption of adiponectin causes insulin resistance and neointimal formation.. J Biol Chem.

[13] Pan WH, Chen JW, Fann C, Jou YS, Wu SY (2000). Linkage analysis with candidate genes: the Taiwan young-onset hypertension genetic study.. Hum Genet.

[14] Tan CE, Ma S, Wai D, Chew SK, Tai ES (2004). Can we apply the National Cholesterol Education Program Adult Treatment Panel definition of the metabolic syndrome to Asians?. Diabetes Care.

[15] Chuang SY, Bai CH, Chen WH, Lien LM, Pan WH (2009). Fibrinogen independently predicts the development of ischemic stroke in a Taiwanese population: CVDFACTS study.. Stroke.

[16] Chen HJ, Bai CH, Yeh WT, Chiu HC, Pan WH (2006). Influence of metabolic syndrome and general obesity on the risk of ischemic stroke.. Stroke.

[17] Pan WH, Hung YT, Shaw NS, Lin W, Lee SD (2005). Elderly Nutrition and Health Survey in Taiwan (1999-2000): research design, methodology and content.. Asia Pac J Clin Nutr.

[18] Hara K, Boutin P, Mori Y, Tobe K, Dina C (2002). Genetic variation in the gene encoding adiponectin is associated with an increased risk of type 2 diabetes in the Japanese population.. Diabetes.

[19] Gu HF, Abulaiti A, Ostenson CG, Humphreys K, Wahlestedt C (2004). Single nucleotide polymorphisms in the proximal promoter region of the adiponectin (APM1) gene are associated with type 2 diabetes in Swedish Caucasians.. Diabetes.

[20] Ohashi K, Ouchi N, Kihara S, Funahashi T, Nakamura T (2004). Adiponectin I164T mutation is associated with the metabolic syndrome and coronary artery disease.. J Am Coll Cardiol.

[21] Arita Y, Kihara S, Ouchi N (1999). Paradoxical decrease of an adipose-specific protein, adiponectin, in obesity.. Biochem Biophys Res Commun.

[22] Zeger SL, Liang KY (1986). Longitudinal data analysis for discrete and continuous outcomes.. Biometrics.

[23] Heid IM, Henneman P, Hicks A, Coassin S, Winkler T (2010). Clear detection of ADIPOQ locus as the major gene for plasma adiponectin: results of genome-wide association analyses including 4659 European individuals.. Atherosclerosis.

[24] Chen W, Srinivasan SR, Elkasabany A, Berenson GS (1999). Cardiovascular risk factors clustering features of insulin resistance syndrome (Syndrome X) in a biracial (Black-White) population of children, adolescents, and young adults: the Bogalusa Heart Study.. Am J Epidemiol.

[25] Austin MA, Edwards KL, McNeely MJ, Chandler WL, Leonetti DL Heritability of multivariate factors of the metabolic syndrome in nondiabetic Japanese americans.. Diabetes.

[26] Agarwal A, Williams GH, Fisher ND (2005). Genetics of human hypertension.. Trends Endocrinol Metab.

[27] Wellcome Trust Case Control Consortium (2007). Genome-wide association study of 14,000 cases of seven common diseases and 3,000 shared controls.. Nature.

[28] Filippi E, Sentinelli F, Trischitta V, Romeo S, Arca M (2004). Association of the human adiponectin gene and insulin resistance.. Eur J Hum Genet.

[29] Mohammadzadeh G, Zarghami N (2009). Associations between single-nucleotide polymorphisms of the adiponectin gene, serum adiponectin levels and increased risk of type 2 diabetes mellitus in Iranian obese individuals.. Scand J Clin Lab Invest.

[30] Iacobellis G, Petrone A, Leonetti F, Buzzetti R (2006). Left ventricular mass and +276 G/G single nucleotide polymorphism of the adiponectin gene in uncomplicated obesity.. Obesity (Silver Spring).

[31] Qi L, Li T, Rimm E, Zhang C, Rifai N (2005). The +276 polymorphism of the APM1 gene, plasma adiponectin concentration, and cardiovascular risk in diabetic men.. Diabetes.

[32] Menzaghi C, Ercolino T, Salvemini L, Coco A, Kim SH (2004). Multigenic control of serum adiponectin levels: evidence for a role of the APM1 gene and a locus on 14q13.. Physiol Genomics.

[33] Jee SH, Sull JW, Lee JE, Shin C, Park J (2010). Adiponectin concentrations: a genome-wide association study.. Am J Hum Genet.

[34] Gable DR, Hurel SJ, Humphries SE (2006). Adiponectin and its gene variants as risk factors for insulin resistance, the metabolic syndrome and cardiovascular disease.. Atherosclerosis.

